# Supplementary material for: Population genetics of swamp eel in the Yangtze River: comparative analyses between mitochondrial and microsatellite data provide novel insights
Source: PeerJ. 2020 Jan 21;8:e8415. doi: 10.7717/peerj.8415 (PMC6979408; doi:10.7717/peerj.8415)
Supplement: Table S1 [file peerj-08-8415-s002.docx]

**Table S1. Sampling information of each site in this study**

| Sample sites | Abbreviation | Sampling basin | Sampling number | Sampling coordinates | |
| --- | --- | --- | --- | --- | --- |
|  |  |  |  | Longitude | Latitude |
| Dang Tu | DT | The tributary of Yangtze River | 30 | 118.62 | 31.52 |
| Wu Wei | WW | The mainstream of Yangtze River | 30 | 117.98 | 31.16 |
| Fan Chang | FC | The tributary of Yangtze River | 30 | 118.24 | 31.11 |
| Gui Chi | GC | The mainstream of Yangtze River | 30 | 117.65 | 30.76 |
| Huai Ning | HN | The tributary of Yangtze River | 30 | 117.03 | 30.75 |
| Wang Jiang | WJ | The mainstream of Yangtze River | 30 | 116.89 | 30.35 |
