# Supplementary material for: Population genetics of swamp eel in the Yangtze River: comparative analyses between mitochondrial and microsatellite data provide novel insights
Source: PeerJ. 2020 Jan 21;8:e8415. doi: 10.7717/peerj.8415 (PMC6979408; doi:10.7717/peerj.8415)
Supplement: Table S2 [file peerj-08-8415-s003.docx]

**Table S2. The primer information of two mitochondrial genes and eight microsatellite loci used in this study**

| Marker name | Primer sequence | Repeat motif | P (HWE) |
| --- | --- | --- | --- |
| *COI* | F: AACCACAAAGACATTGGCACC | - | - |
|  | R: CCAAAGCCTGGAAGAATCAAG |  |  |
| *Cyt b* | F: GCCCTACGAAAACGACAT | - | - |
|  | R: CTTACAACGCCGATGCT |  |  |
| *Ma 73* | F: CATTTCTCAGATTCACCCA | (CA)_18_ | 1.00 |
|  | R: GTATGAGCCACTGAAGAGG |  |  |
| *Ma 32* | F: GAGGTCGTTCAGTTCAAT | (CA)_18_ | 0.82 |
|  | R: AACAAAACAGGTGGACAGA |  |  |
| *Ma 59* | F: CTCAGTGATTCCAGTGCC | (AC)_21_ | 0.72 |
|  | R: CGCACTGCTTTCTAAACT |  |  |
| *Ma 15* | F: CACCTCCCTGCTGGAATGT | (TG)_21_ | 0.95 |
|  | R: GTCGTACACAGCCTTGCC |  |  |
| *Ma 74* | F: ACTGCCATGTCCACTAAG | (CA)_13_ | 0.41 |
|  | R: GGTCTATACAGACAGAGGCT |  |  |
| *Ma72* | F: AGTTCAGGTGAACATGCA | (GA)_12_(GC)_4_(GT)_10_ | 0.89 |
|  | R: GCAGCAGGGGATATTGAG |  |  |
| *Mal 06* | F: TATCAGTGGATCTGCCACCA | (GT)_18_ | 0.75 |
|  | R: CAGGAGAGTCACAGCCATAAA |  |  |
| *Mal 007* | F: CATCAGGGCTAAAGAAAATGTCCA | (TG)_16_ | 0.18 |
|  | R: CCAGCCATCAGTCTGAGAAATCC |  |  |
