# Supplementary material for: Population genetics of swamp eel in the Yangtze River: comparative analyses between mitochondrial and microsatellite data provide novel insights
Source: PeerJ. 2020 Jan 21;8:e8415. doi: 10.7717/peerj.8415 (PMC6979408; doi:10.7717/peerj.8415)
Supplement: Table S3 [file peerj-08-8415-s004.docx]

**Table S3. The genetic (below diagonal) and geographic (above diagonal) (km) distance matrixes.**

|  | DT | WW | FC | GC | HN | WJ |
| --- | --- | --- | --- | --- | --- | --- |
| DT |  | 92.7 | 101.6 | 167 | 237.9 | 264.9 |
| WW | 0.7839 |  | 58.5 | 78.9 | 149.8 | 168.8 |
| FC | 0.7075 | 0.4309 |  | 132.8 | 203.7 | 230.7 |
| GC | 0.8605 | 0.1878 | 0.4540 |  | 70.9 | 97.9 |
| HN | 0.9121 | 0.4723 | 0.5921 | 0.4763 |  | 92.8 |
| WJ | 0.7628 | 0.1388 | 0.4079 | 0.1133 | 0.3732 |  |
